# Programmed −2/−1 Ribosomal Frameshifting in Simarteriviruses: an Evolutionarily Conserved Mechanism

**DOI:** 10.1128/JVI.00370-19

**Published:** 2019-07-30

**Authors:** Yanhua Li, Andrew E. Firth, Ian Brierley, Yingyun Cai, Sawsan Napthine, Tao Wang, Xingyu Yan, Jens H. Kuhn, Ying Fang

**Affiliations:** a Department of Diagnostic Medicine and Pathobiology, Kansas State University, Manhattan, Kansas, USA; b Division of Virology, Department of Pathology, University of Cambridge, Cambridge, United Kingdom; c Integrated Research Facility at Fort Detrick, National Institute of Allergy and Infectious Diseases, National Institutes of Health, Frederick, Maryland, USA; d Yangzhou University, Yangzhou, People’s Republic of China; University of Texas Southwestern Medical Center

**Keywords:** −2/−1 programmed ribosomal frameshifting, arterivirus, simarterivirus

## Abstract

The −2/−1 programmed ribosomal frameshifting (−2/−1 PRF) mechanism in porcine reproductive and respiratory syndrome virus (PRRSV) leads to the translation of two additional viral proteins, nonstructural protein 2TF (nsp2TF) and nsp2N. This −2/−1 PRF mechanism is transactivated by a viral protein, nsp1β, and cellular poly(rC) binding proteins (PCBPs). Critical elements for −2/−1 PRF, including a slippery sequence and a downstream C-rich motif, were also identified in 11 simarteriviruses. However, the slippery sequences (XXXUCUCU instead of XXXUUUUU) in seven simarteriviruses can only facilitate −2 PRF to generate nsp2TF. The nsp1β of simian hemorrhagic fever virus (SHFV) was identified as a key factor that transactivates both −2 and −1 PRF, and the universally conserved Tyr111 and Arg114 in nsp1β are essential for this activity. *In vitro* translation experiments demonstrated the involvement of PCBPs in simarterivirus −2/−1 PRF. Using SHFV reverse genetics, we confirmed critical roles of nsp1β, slippery sequence, and C-rich motif in −2/−1 PRF in SHFV-infected cells. Attenuated virus growth ability was observed in SHFV mutants with impaired expression of nsp2TF and nsp2N. Comparative genomic sequence analysis showed that key elements of −2/−1 PRF are highly conserved in all known arteriviruses except equine arteritis virus (EAV) and wobbly possum disease virus (WPDV). Furthermore, −2/−1 PRF with SHFV PRF signal RNA can be stimulated by heterotypic nsp1βs of all non-EAV arteriviruses tested. Taken together, these data suggest that −2/−1 PRF is an evolutionarily conserved mechanism employed in non-EAV/-WPDV arteriviruses for the expression of additional viral proteins that are important for viral replication.

**IMPORTANCE** Simarteriviruses are a group of arteriviruses infecting nonhuman primates, and a number of new species have been established in recent years. Although these arteriviruses are widely distributed among African nonhuman primates of different species, and some of them cause lethal hemorrhagic fever disease, this group of viruses has been undercharacterized. Since wild nonhuman primates are historically important sources or reservoirs of human pathogens, there is concern that simarteriviruses may be preemergent zoonotic pathogens. Thus, molecular characterization of simarteriviruses is becoming a priority in arterivirology. In this study, we demonstrated that an evolutionarily conserved ribosomal frameshifting mechanism is used by simarteriviruses and other distantly related arteriviruses for the expression of additional viral proteins. This mechanism is unprecedented in eukaryotic systems. Given the crucial role of ribosome function in all living systems, the potential impact of the in-depth characterization of this novel mechanism reaches beyond the field of virology.

## Abstract (precis)

Simarteriviruses are a group of arteriviruses infecting nonhuman primates, and a number of new species have been established in recent years. Although these arteriviruses are widely distributed among African nonhuman primates of different species, and some of them cause lethal hemorrhagic fever disease, this group of viruses has been undercharacterized. Since wild nonhuman primates are historically important sources or reservoirs of human pathogens, there is concern that simarteriviruses may be preemergent zoonotic pathogens. Thus, molecular characterization of simarteriviruses is becoming a priority in arterivirology. In this study, we demonstrated that an evolutionarily conserved ribosomal frameshifting mechanism is used by simarteriviruses and other distantly related arteriviruses for the expression of additional viral proteins. This mechanism is unprecedented in eukaryotic systems. Given the crucial role of ribosome function in all living systems, the potential impact of the in-depth characterization of this novel mechanism reaches beyond the field of virology.

## INTRODUCTION

The family *Arteriviridae* within the order *Nidovirales* initially included four positive-stranded RNA viruses, namely, porcine reproductive and respiratory syndrome virus (PRRSV), mouse lactate dehydrogenase-elevating virus (LDV), equine arteritis virus (EAV), and simian hemorrhagic fever virus (SHFV), which were assigned to four species (1). Currently, this family has been reclassified into six subfamilies with 19 species (2), in which the two genotypes of PRRSV currently belong to two different species (PRRSV-1 and PRRSV-2), and the newly identified wobbly possum disease virus (WPDV) (3), Chinese rat arterivirus (RatAV), and Ningxia rat arterivirus (RatAV_Ningxia2015) (2, 4), African pouched rat arterivirus (APRAV) (5), and new simarteriviruses were added as members of new species (6). Among these arteriviruses, EAV (*Equarterivirinae*) and PRRSV-1 and PRRSV-2 (*Variarterivirinae*) are economically important veterinary pathogens (7).

Simarteriviruses comprise a rapidly expanding subfamily of undercharacterized arteriviruses now known to infect a wide range of nonhuman primates. The most notorious simarteriviruses, Pebjah virus (PBJV), simian hemorrhagic encephalitis virus (SHEV), and SHFV, repeatedly caused highly lethal hemorrhagic fever epizootics among captive macaques in the United Kingdom, United States, and the Soviet Union throughout the 1960s to 1990s (reviewed in reference 8), but their natural host reservoirs are unknown. In contrast, other simarteriviruses found in nature, specifically, those in subclinically infected African nonhuman primates, have not been implicated in epizootics. These viruses include De Brazza’s monkey virus 1 (DeBMV-1) in De Brazza’s monkeys (Cercopithecus neglectus), Drakensberg Mountain vervet virus (DMVV-1) in vervet monkeys (Chlorocebus pygerythrus) (6), Kafue kinda-chacma baboon virus (KKCBV) in Kinda baboons (Papio kindae), Kibale red colobus viruses 1 and 2 (KRCV-1 and KRCV-2) in Ugandan red colobus (Procolobus rufomitratus tephrosceles) (9), Kibale red-tailed guenon viruses 1 and 2 (KRTGV-1/2) in red-tailed monkeys (Cercopithecus ascanius) (10), Mikumi yellow baboon virus 1 (MYBV-1) in yellow baboons (Papio cynocephalus) (11), southwest baboon virus 1 (SWBV-1) in olive baboons (Papio anubis) (11), and Zambian malbrouck virus 1 (ZMbV-1) in malbrouck monkeys (Chlorocebus cynosuros) (6). In experimental settings, KRCV-1 can cause mild disease in crab-eating macaques (Macaca fascicularis) (12), whereas SWBV-1 infects but does not appear to cause disease in rhesus monkeys (Macaca mulatta) (13). Since wild nonhuman primates are historically important sources/reservoirs of human pathogens and because of their broad and diverse distribution among African monkeys, there is concern that some simian arteriviruses may be preemergent zoonotic pathogens (14). Hence, increased molecular characterization of simarteriviruses is becoming a priority in arterivirology.

Most RNA viruses have polycistronic genomes and have evolved strategies to overcome a limitation of the eukaryotic translation apparatus, namely that in general, only the 5′-most open reading frame (ORF) on an mRNA is translated. These include noncanonical translation mechanisms, such as programmed ribosomal frameshifting (PRF) and alternative initiation, and in addition, the expression of polyproteins that are subsequently cleaved by viral or host proteases. Viruses may also produce subgenomic mRNAs that are functionally monocistronic. Arteriviruses use several of these strategies to coordinate their complex replication cycles (15, 16).

Arterivirus genomes vary in length between 12.5 and 15.5 kb and contain 10 to 15 known ORFs. All but two are located toward the 3′ end of the genome and encode viral structural proteins that are translated from a nested set of subgenomic mRNAs (17). ORF1a and ORF1b, at the 5′ end of the genome, comprise some three-quarters of the coding capacity and encode replicase-associated proteins. Translation of the genomic RNA yields the ORF1a polyprotein and, in addition, an ORF1ab fusion polyprotein following −1 PRF at the ORF1a/ORF1b junction (18, 19). The two polyproteins are processed into individual functional nonstructural proteins (nsps) by ORF1a-encoded protease domains. In PRRSV, these comprise two papain-like proteases, PLP1α and PLP1β, located in nsp1α and nsp1β, respectively, a papain-like protease (PLP2) domain situated in the N terminus of nsp2, and a serine protease domain residing in nsp4. The rapid release of nsp1α, nsp1β, and nsp2 from the N terminus of the polyprotein is mediated by autocatalytic cleavage with PLP1α (between nsp1α and nsp1β [nsp1α/1β]), PLP1β (nsp1β/2), and PLP2 (nsp2/3) (20). In the SHFV nsp1 region, three papain-like proteases (PLPα, PLPβ, and PLPγ) are present within nsp1 subunits nsp1α, nsp1β, and nsp1γ. Similar to the case for PRRSV, these nsp1 subunits are released from the N terminus of SHFV polyproteins by autocleavages (21). Nsp2, the largest replicase subunit of arteriviruses, is a multifunctional protein that plays important roles in viral replication and virus-host interaction (20, 22–30). In addition to cleavage of the nsp2/3 site, the PLP2 domain functions as a cofactor for the serine protease during proteolytic processing of the C-terminal region of pp1a and pp1ab (20, 31). The C terminus of nsp2 contains a highly conserved Cys-rich domain of unknown function and a multispanning transmembrane domain that plays a critical role in the formation of membranous structures (32).

PRRSV uses an unusual −2 PRF signal to direct efficient expression of an additional protein from the +1 reading frame overlapping the nsp2-encoding region. The −2 PRF generates a transframe (TF) fusion protein, nsp2TF. It consists of the N-terminal two-thirds of nsp2, followed by a unique C-terminal domain that is encoded by a novel overlapping TF ORF (33). At the same frameshifting site, an immediate stop codon is generated by −1 PRF, which leads to the expression of a truncated nsp2, designated nsp2N (34). Remarkably, both cellular poly(rC) binding proteins (PCBPs) and viral nsp1β are required for efficient −2/−1 PRF (35). Sequence analysis shows that the signals for −2/−1 PRF, including a slippery sequence and downstream C-rich RNA motif, are highly conserved in all arterivirus genomes except that of EAV (36). However, variations in slippery sequences have been identified in several newly identified simarteriviruses. In this study, we demonstrate that −2/−1 PRF identified in PRRSV is evolutionarily conserved in non-EAV/-WPDV arteriviruses, with particular emphasis on simarteriviruses. This study provides additional insights into the biological characteristics of arteriviruses and advances our knowledge of noncanonical translation mechanisms in both virus infection and cellular systems.

## RESULTS

### Key elements of a −2/−1 PRF mechanism are conserved in non-EAV/-WPDV arteriviruses.

**FIG 1:**
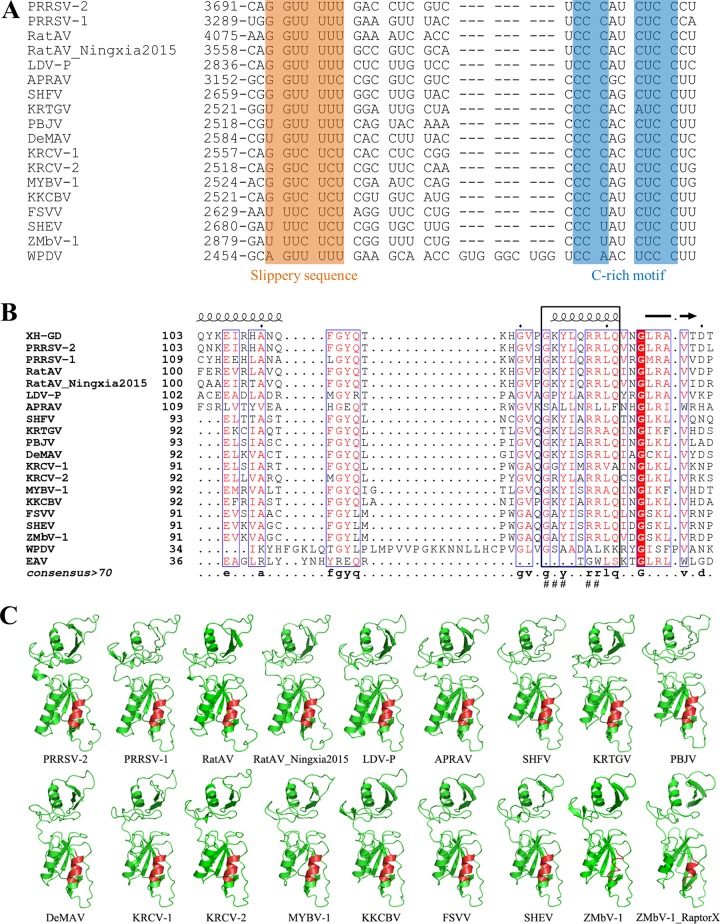
Bioinformatics analysis of −2/−1 programmed ribosomal frameshifting (PRF) RNA signals and nsp1βs of arteriviruses. (A) The −2/−1 PRF signals, including the slippery sequence and downstream C-rich RNA motif, identified in the nsp2 coding region of arteriviruses. For each sequence, the genome coordinate of the first nucleotide in the alignment is specified. (B) A highly conserved α-helix motif in the papain-like cysteine protease β domain (PCPβ) of arterivirus nsp1β. The protein sequences of arterivirus nsp1βs were predicted as described previously (36). Sequence alignment of arterivirus nsp1βs was performed with Clustal Omega (56), and the figure was created with ESPript 3 (57). The highly conserved α-helix motif is indicated with a rectangle in black, and the residues in this motif targeted for mutagenesis are marked with hash signs (#). For each sequence, the nsp1β or nsp1 coordinate of the first amino acid in the alignment is specified. (C) The 3-D structures of arterivirus nsp1βs predicted by the I-TASSER server (40). The α-helix secondary structure of the highly conserved motif in ZMbV-1 nsp1β was also predicted, using the RaptorX server (41), and is identified as ZMbV-1_RaptorX. Representative virus strains of 19 arteriviruses were included in the above-described bioinformatics analysis (2); their GenBank accession numbers are as follows: XH-GD (EU624117.1), PRRSV-2 (SD95-21; KC469618.1), PRRSV-1 (SD01-08; DQ489311.1), RatAV (KP280006.1), RatAV_Ningxia2015 (KU302440.1), LDV-P (U15146.1), APRAV (NC_026439.1), SHFV (KM373784.1), KRTGV (JX473847.1), PBJV (KR139838.1), DeMAV (KP126831.1), KRCV-1 (HQ845737.1), KRCV-2 (KC787631.1), MYBV-1 (KM110935.1), KKCBV (KT447550.1), FSVV (KR862306.1), SHEV (KM677927.1), ZMbV-1 (KT166441.1), EAV (X53459.3), and WPDV (JN116253.3). The highly conserved α-helix motif is highlighted in red.

Previously, we identified the frameshift RNA signal of PRRSV, which consists of a slippery sequence (G_GUU_UUU) and a downstream C-rich motif (CCCANCUCC) that is separated from the slippery sequence by a 10-nucleotide (nt) spacer (34, 35). In addition, the PRRSV replicase subunit nsp1β functions as a transactivator for both −2 and −1 programmed ribosomal frameshifting (PRF); in particular, a highly conserved α-helix motif in nsp1β is critical for PRF transactivation (34). In this study, comparative genomic sequence analysis of arteriviruses was performed to identify the key elements of −2/−1 PRF in viruses of all relevant species in the *Arteriviridae* family. The slippery sequence and C-rich motif of the PRF signal were identified in all simarteriviruses, although a few substitutions were observed for viruses of different species, including U_GUU_UUU (KRTGV, PBJV, and De Brazza’s monkey arterivirus [DeMAV]), G_GUC_UCU (KRCV-1, KRCV-2, MYBV-1, and KKCBV), and U_UUC_UCU (free state vervet virus [FSVV], SHEV, and ZMbV-1) (Fig. 1A). These signals all allow anticodon-codon re-pairing in the A site following a −2 PRF, but as in PRRSV, the potential for re-pairing in the P site is more limited. Of note, these PRF RNA signals were found in all known arteriviruses except EAV. Additionally, the distance between slippery sequence and C-rich motif is 9 or 10 nucleotides (nt), except in WPDV, in which the closest C-rich sequence is at a distance of 19 nt.

The highly conserved motif in PRRSV-2 nsp1β is also found in all known arteriviruses except WPDV and EAV (Fig. 1B). In line with our previous studies in PRRSV (37, 38), two arginine residues in this motif are conserved among most arteriviruses (Arg114 and Arg115 in SHFV). In APRAV-1, the second arginine is replaced with a leucine residue (Fig. 1B). The three-dimensional (3-D) structures of nsp1β of arteriviruses, with the exceptions of WPDV and EAV, were predicted with the I-TASSER server and RaptorX server guided by the known crystal structure of PRRSV-2 (XH-GD strain) nsp1β (Fig. 1C) (39–41). The highly conserved motif consistently forms an α-helix. The longer distance between slippery sequence and C-rich sequence in WPDV and its different nsp1β protein conformation compared to those of other arteriviruses suggest that WPDV likely does not utilize frameshifting at this site. Taken together, these data suggest that −2/−1 PRF might be an evolutionarily conserved mechanism for the expression of additional viral proteins in non-EAV/-WPDV arteriviruses.

### SHFV nsp1β transactivates −2/−1 ribosomal frameshifting.

**FIG 2:**
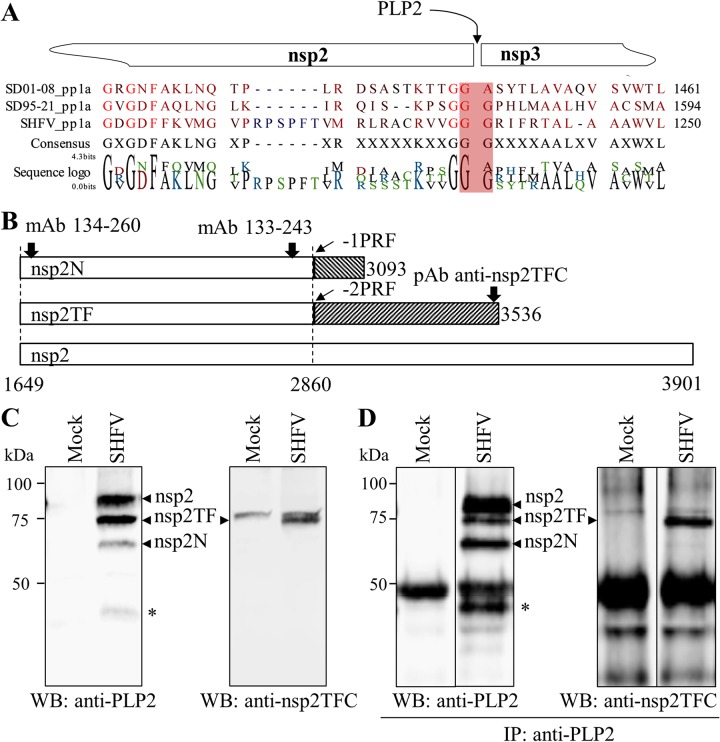
SHFV nsp2-related proteins expressed in virus-infected MARC-145 cells. (A) The C-terminal end of SHFV nsp2s predicted by sequence alignment of polyprotein 1a (pp1a) from PRRSV-1 (SD01-08; DQ489311.1), PRRSV-2 (SD95-21; KC469618.1), and SHFV (KM373784.1). SHFV nsp2 is predicted to be released from pp1a through proteolytic cleavage by the PLP2 domain. The cleavage site between SHFV nsp2 and nsp3 was predicted to be at Gly1236/Gly1237. For each sequence, the pp1a coordinate of the last amino acid in the alignment is specified. (B) Schematic diagram of putative SHFV nsp2, nsp2TF, and nsp2N. SHFV nsp2 is encoded by the SHFV genomic region, comprising nucleotides (nt) 1649 to 3901, whereas nsp2TF (nt 1649 to 2860 + 2859 to 3536) and nsp2N (nt 1649 to 2860 + 2860 to 3093) are translated through −2 PRF and −1 PRF, respectively. The epitopes recognized by antibodies are indicated by black arrows. (C) SHFV nsp2-related proteins detected by Western blot (WB) analysis. MARC-145 cells were infected with SHFV at an MOI of 0.01, and cell lysates were harvested at 36 hpi. The nsp2-related products, including nsp2, nsp2TF, nsp2N, and an unknown nsp2-related protein, marked with an asterisk (*), were detected by anti-PLP2 mAb 134-260. SHFV nsp2TF was also recognized by rabbit pAb against the nsp2TF C-terminal peptide (nsp2TFC). (D) SHFV nsp2-related proteins detected by immunoprecipitation and WB analysis. MARC-145 cells were infected with SHFV at an MOI of 0.01, and cell lysates were harvested at 36 hpi. The nsp2-related products were immunoprecipitated (IP) using anti-PLP2 mAb 133-243 and probed by WB with anti-PLP2 mAb 134-260 and rabbit anti-nsp2TFC pAb. An unknown nsp2-related protein is marked with an asterisk (*).

In PRRSV, −2/−1 PRF results in the translation of two novel proteins, nsp2TF and nsp2N (34). To test our hypothesis that −2/−1 PRF is utilized to translate nsp2TF and nsp2N proteins in simarteriviruses, we initially characterized nsp2-related proteins in SHFV-infected MARC-145 cells. For the detection of nsp2-related proteins, we generated two monoclonal antibodies (mAbs) (mAb 133-243 and mAb 134-260) against the PLP2 domain, which is shared by SHFV nsp2 and the predicted nsp2TF and nsp2N. A rabbit polyclonal antibody (pAb) against the unique epitope (RLDSTVVFEETTPL) at the C terminus of nsp2TF was also generated for specific identification of nsp2TF. The N terminus of SHFV nsp2 (Gly484/Gly485/Gly486; the residue positions are based on the SHFV pp1a sequence) was previously determined by mass spectrometry sequence analysis (21). We further predicted the C terminus of SHFV nsp2 based on the putative cleavage site between nsp2 and nsp3. As shown in Fig. 2A, the cleavage site of SHFV nsp2/3 was predicted to be between Gly1236 and Gly1237 (the residue positions based on SHFV pp1a sequence) by analysis of sequence alignment with the nsp2/3 cleavage site in PRRSVs (20). Thus, the SHFV nsp2 gene is predicted to be nucleotides 1649 to 3901 of the SHFV genome, and the predicted molecular mass of the protein is 81.2 kDa. If −2/−1 PRF occurs, nsp2TF is translated from SHFV genomic nucleotides 1649 to 2860 fused to nucleotides 2859 to 3536, which results in a product with a predicted molecular mass of 68.7 kDa, and nsp2N is translated from SHFV genomic nucleotides 1649 to 2860 fused to nucleotides 2860 to 3093, resulting in a product with a predicted molecular mass of 52 kDa (Fig. 2B).

Western blot analysis was performed to identify nsp2-related proteins in SHFV-infected MARC-145 cells. As shown by the results in Fig. 2, four major virus-specific bands were detected by mAb 134-260 against SHFV PLP2 (Fig. 2C, left). The top three largest proteins appear to be nsp2, nsp2TF, and nsp2N, although their masses are larger than those that were predicted—a phenomenon that was previously reported for nsp2-related proteins of PRRSV (33). The rabbit polyclonal antibody specifically designed to detect SHFV nsp2TF recognized two protein bands in the Western blot analysis. The top band appeared to be a cellular protein, since this band was also detected in mock-infected cells, while the second large protein band was specific to nsp2TF (Fig. 2C, right), indicating that SHFV indeed expresses nsp2TF via −2 PRF. The fourth band, with a size of less than 50 kDa, is a C-terminally truncated isoform of nsp2, which may be generated by proteolytic cleavage by PLP2 or some other proteinase. The identity of this product needs to be further studied in the future. To confirm the identity of these nsp2-related proteins, immunoprecipitation (IP) was performed with anti-PLP2 mAb 133-243 using cell lysate of SHFV-infected MARC-145 cells, whereas cell lysate of mock-infected MARC-145 cells was used as a negative control. As expected, four major bands were detected in the IP product using mAb 134-260 (Fig. 2D, left), and the second-largest protein band was also recognized by the nsp2TF-specific pAb (Fig. 2D, right).

**FIG 3:**
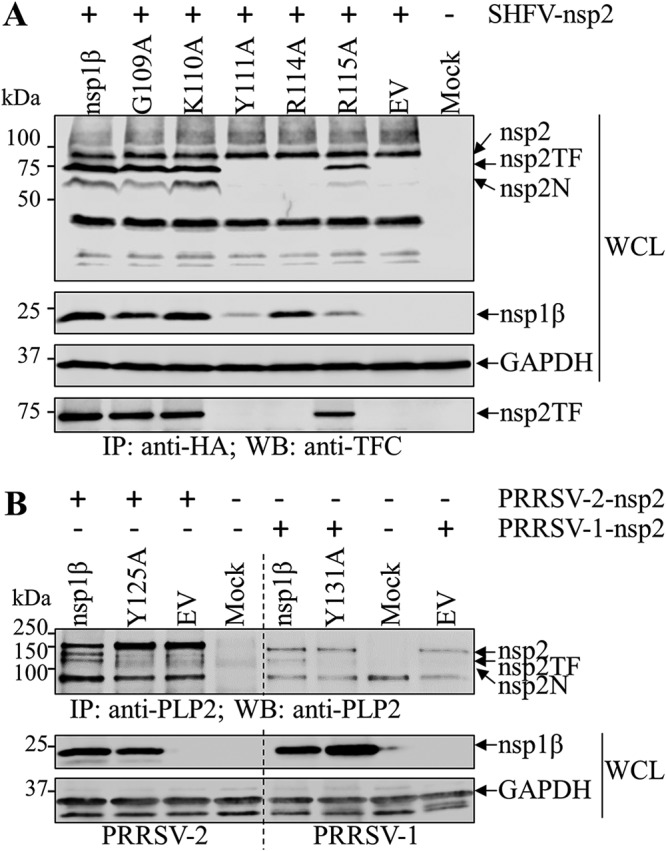
Identification of key residues involved in the PRF transactivation function of nsp1β from SHFV and PRRSV. (A) Immunoprecipitation and Western blot (WB) analysis of amino acid residues critical for the transactivation function of SHFV nsp1β. HEK-293T cells were cotransfected with a plasmid expressing HA-tagged SHFV nsp2 and a plasmid expressing FLAG-tagged SHFV nsp1β or mutants thereof. An empty vector (EV) was used as the control. Whole-cell lysates (WCLs) were harvested at 36 h posttransfection (hpt). The expression of nsp2, nsp2TF, and nsp2N was detected by WB using anti-HA mAb, whereas FLAG-tagged nsp1β was detected using anti-FLAG mAb M2. The expression of HA-tagged nsp2TF was further confirmed by IP using anti-HA mAb and WB detection using anti-nsp2TF C-terminal peptide pAb (anti-TFC). (B) Immunoprecipitation and WB analysis of amino acid residues critical for the transactivation function of nsp1β from PRRSV-1 and PRRSV-2. HEK-293T cells were cotransfected with a plasmid expressing PRRSV nsp2 and a plasmid expressing FLAG-tagged PRRSV nsp1β or mutants thereof. An empty vector (EV) was used as a control. PRRSV nsp2-related proteins were immunoprecipitated with anti-PRRSV PLP2 mAb. The expression of nsp2, nsp2TF, and nsp2N was evaluated by WB using anti-PRRSV PLP2 domain mAb, and FLAG-tagged nsp1β was detected with anti-FLAG mAb M2. GAPDH was monitored as a loading control.

An ectopic expression system was further employed to investigate whether the expression of SHFV nsp2TF and nsp2N requires nsp1β as a PRF transactivator. In HEK-293T cells transfected with a plasmid expressing SHFV nsp2 alone, the PRF products, nsp2TF and nsp2N, were not detected (Fig. 3A, lane marked “EV”). When HEK-293T cells were cotransfected with plasmids expressing nsp2 and nsp1β, both PRF products, nsp2TF and nsp2N, were detected (Fig. 3A, lane marked “nsp1β”). These results indicate that SHFV nsp1β is critical for the expression of nsp2TF and nsp2N. To further identify the key residues in SHFV nsp1β, amino acids Gly109, Lys110, Tyr111, Arg114, and Arg115 in the conserved motif (Fig. 1A) were targeted for mutagenesis with alanine replacement. Consistent with our previous findings on PRRSV (34, 38), mutation of R to A at position 114 (R114A) completely abolished the expression of nsp2TF and nsp2N, whereas the G109A and K110A mutations had no obvious effects on the expression of nsp2TF and nsp2N. The R115A mutant stimulated lower expression levels of nsp2TF and nsp2N, which may be due to the lower expression level of this nsp1β mutant. The Y111A mutant was also unable to stimulate the expression of nsp2TF and nsp2N, indicating that Tyr111 is another key residue for nsp1β’s function in transactivation of −2/−1 PRF (Fig. 3A). This result was further confirmed with PRRSV: nsp2TF and nsp2N were not detectable in HEK-293T cells expressing PRRSV nsp1β mutants that contained the corresponding alanine substitution at Y125 (PRRSV-2) or Y131 (PRRSV-1) (Fig. 3B). Similar to the R115A mutant, a lower expression level was observed for the SHFV Y111A mutant (Fig. 3A). However, this lower expression should not be the direct reason leading to the loss of expression of nsp2TF and nsp2N, since the Y111A mutant, with an expression level similar to that of wild-type (WT) nsp1β in vY111A-infected cells, was unable to stimulate the expression of nsp2TF and nsp2N (see Fig. 5).

**FIG 4:**
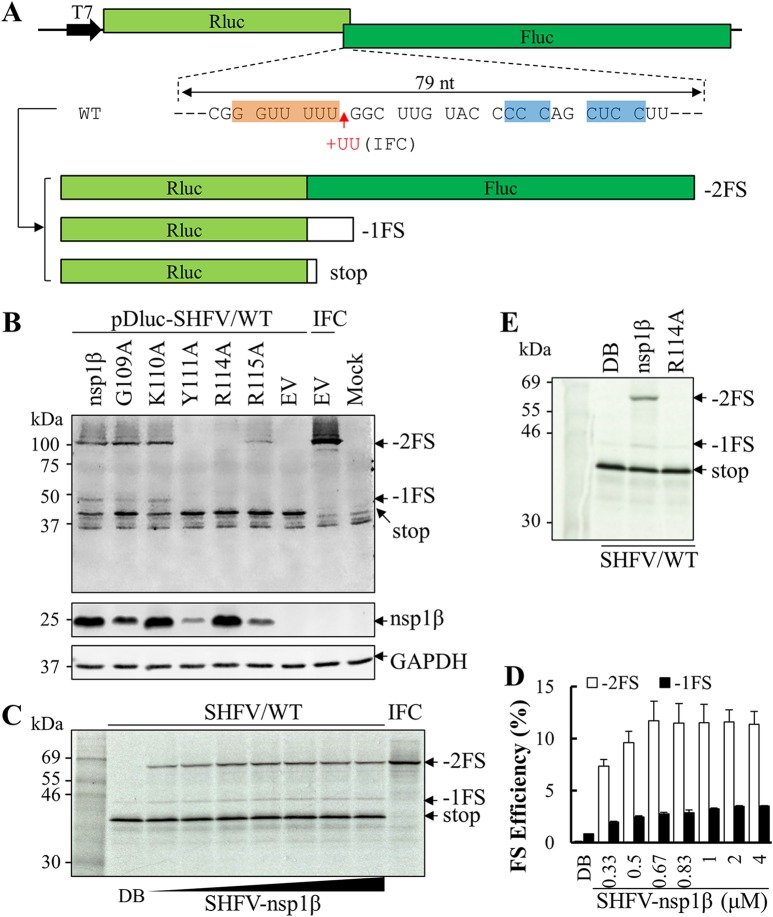
SHFV nsp1β stimulates −2/−1 PRF in pDluc reporter systems. (A) Schematic diagram of dual luciferase (pDluc) constructs. A 79-nt sequence (WT) containing the SHFV putative slippery sequence and downstream C-rich motif was inserted between *Renilla* luciferase (Rluc) and firefly luciferase (Fluc; in the −2 frame relative to Rluc) ORFs. The in-frame control (IFC) construct was generated by inserting two Us after the slippery sequence. In addition to the stop product translated without frameshifting, −2FS and −1FS products containing *Renilla* luciferase could be translated via −2 PRF and −1 PRF, respectively. (B) Identification of critical residues in SHFV nsp1β involved in −2/−1 PRF. HEK-293T cells were cotransfected with plasmid pDluc-SHFV/WT containing wild-type SHFV −2/−1 PRF signal and a plasmid expressing SHFV nsp1β or mutants thereof; empty vector (EV) was used as the control. Nonframeshift, −1 PRF, and −2 PRF products were detected by Western blotting using anti-*Renilla* luciferase (Rluc) mAb and are indicated as stop, −1FS, and −2FS, respectively. FLAG-tagged nsp1β was detected with mAb M2, and GAPDH was detected as a loading control. (C) SHFV/WT mRNA transcribed from plasmid pDluc-SHFV/WT was translated in rabbit reticulocyte lysate (RRL) in the presence of different concentrations of GST-tagged nsp1β (μM) or with dilution buffer (DB) as a control. (D) Efficiencies of −2 PRF and −1 PRF were calculated based on the quantified bands using ImageQuant TL software (GE Healthcare). Bars and error bars show mean values and standard errors of the means (SEM). (E) *In vitro* translation of SHFV/WT mRNA was performed with 1 μM GST-nsp1β or its mutant. (C and E) *In vitro* translation products were resolved in 12% SDS-PAGE and visualized by autoradiography. Products generated without frameshifting or from −1 or −2 PRF are indicated as stop, −1FS, and −2FS, respectively.

A dual luciferase reporter system was employed to confirm the activity of SHFV nsp1β in stimulating −2/−1 PRF at the predicted PRF signal in the SHFV genome. A dual luciferase reporter plasmid, pDluc-SHFV/WT, was generated using the approach that we described previously (34), in which 79 nt containing the SHFV PRF signal was inserted in the plasmid between its *Renilla* luciferase (Rluc) and firefly luciferase (Fluc) ORFs. In this construct, Fluc was encoded in the −2 frame relative to the upstream Rluc ORF. Besides an in-frame Rluc product (stop), two frameshifting products (−2FS and −1FS) could be expressed through −2/−1 PRF (Fig. 4A). The predicted molecular masses of the stop, −1FS, and −2FS products are 40.4 kDa, 43.4 kDa, and 100.1 kDa, respectively. In HEK-293T cells cotransfected with the reporter plasmid and a plasmid expressing SHFV nsp1β, all three nsp2-related proteins were detected at the predicted sizes by a mAb against their shared N-terminal Rluc (Fig. 4B, lane marked “nsp1β”). Only the in-frame translation product (stop) was detected in cells without the expression of SHFV nsp1β (Fig. 4B, lane marked “EV” in the pDluc-SHFV/WT-transfected cells). The ability of nsp1β mutants to activate −2/−1 PRF was also tested using this reporter system. In cells expressing the SHFV nsp1β Y111A or R114A mutants, −2FS and −1FS expression products were undetectable, indicating that Tyr111 and Arg114 are essential to nsp1β’s ability to stimulate frameshifting (Fig. 4B). In cells expressing the SHFV nsp1β R115A mutant, reduced expression of the −2FS product was observed, whereas the −1FS product was undetectable. Compared to the reduced expression levels of nsp2TF and nsp2N stimulated by the R115A mutant in Fig. 3A, SHFV nsp1β R115A mutant in the luciferase reporter system may stimulate a much lower level of −1FS, which is under the detection limit of Western blot analysis.

These findings were confirmed in an *in vitro* translation assay using rabbit reticulocyte lysate (RRL) programmed with an *in vitro*-transcribed reporter mRNA and purified nsp1β protein (Fig. 4C). In this system, the predicted masses of stop, −1FS, and −2FS products are 40.4 kDa, 43.4 kDa, and 70.8 kDa. When *in vitro* translation was performed with only reporter mRNA from pDluc-SHFV/WT, the −2FS product was not detected and only a trace amount of −1FS was observed (Fig. 4C, lane marked “DB” [dilution buffer]). In contrast, with the addition of purified nsp1β, −1FS and −2FS were detected at the predicted molecular masses. Furthermore, within the range of 0∼1 μM of nsp1β, the expression levels of the −1 and −2 PRF products were observed to be dose dependent (Fig. 4C and D). When the concentration of nsp1β was higher than 1 μM, both frameshifts reached their maximum efficiencies, which were 11.7% (−2 PRF) and 3.5% (−1 PRF) (Fig. 4D). Furthermore, the purified nsp1β R114A mutant was impaired in its ability to stimulate −2/−1 PRF, as demonstrated by the disappearance of the −2FS product, and reduction of −1FS product to the background level (Fig. 4E).

**FIG 5:**
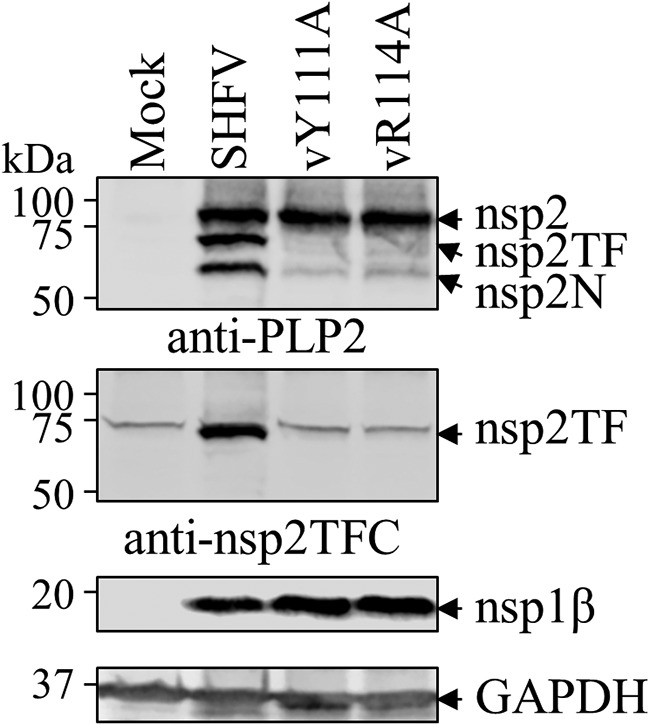
Tyr111 and Arg114 residues on SHFV nsp1β are critical for −2/−1 PRF during SHFV infection. MARC-145 cells were infected with SHFV or mutants (vY111A and vR114A) at an MOI of 0.01, and cell lysates were harvested at 36 hpi. The nsp2-related products were detected by anti-PLP2 mAb 134-260. SHFV nsp2TF was also recognized by rabbit pAb against the nsp2TF C-terminal peptide (nsp2TFC). The expression of SHFV nsp1β and mutants thereof was evaluated with mAb 76-69. GAPDH was monitored as a loading control.

To further confirm these results in the context of SHFV infection, two recombinant viruses, vY111A and vR114A, were rescued using an SHFV reverse genetics system (constructed from variant strain NIH LVR42-0/M6941). The ability of vY111A and vR114A to express nsp2TF and nsp2N was evaluated by Western blot analysis using cell lysates of SHFV-infected MARC-145 cells. In MARC-145 cells infected with either of the two nsp1β mutants, nsp2TF was not detected and only a low expression level of nsp2N was detected (Fig. 5), which is consistent with the results generated in the *in vitro* expression systems.

### Both the slippery sequence and C-rich motif are required for efficient −2/−1 PRF in simian arteriviruses.

**FIG 6:**
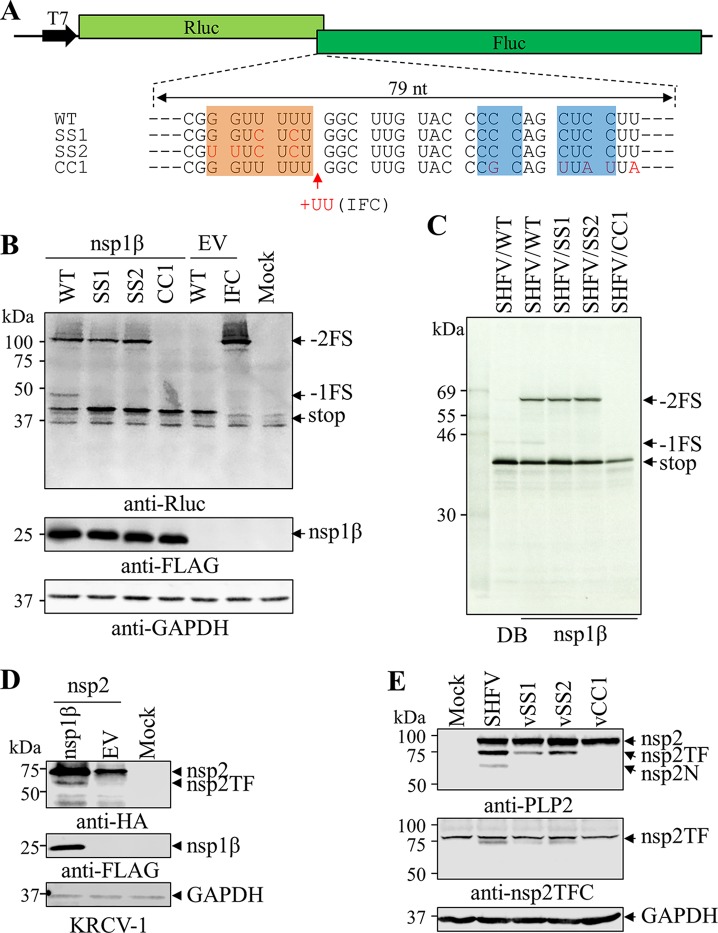
The slippery sequence and downstream C-rich RNA motif are required for SHFV −2/−1 PRF. (A) Schematic diagram of dual luciferase (pDluc) constructs. A 79-nt sequence (WT) containing the SHFV putative slippery sequence and downstream C-rich motif assumed to be necessary for −2/−1 PRF was inserted between *Renilla* luciferase (Rluc) and firefly luciferase (Fluc; in the −2 frame relative to Rluc) ORFs. Constructs SS1 and SS2 contain substitutions (red) at the slippery sequence to mimic natural PRF signal variations identified in simarteriviruses other than SHFV. The CC1 construct contains substitutions (red) to disrupt the C-rich motif, while the in-frame control (IFC) construct was generated by inserting two Us after the slippery sequence. (B) Effect of mutations in the slippery sequence (SS1 and SS2) or C-rich motif (CC1) on SHFV −2/−1 PRF in the pDluc reporter system. HEK-293T cells were cotransfected with pDluc-SHFV/WT or mutants thereof and a plasmid expressing SHFV nsp1β; empty vector (EV) was used as a control. Nonframeshift, −1 PRF, and −2 PRF products (indicated as stop, −1FS, and −2FS, respectively) were detected by Western blotting using anti-*Renilla* luciferase (Rluc) mAb. FLAG-tagged nsp1β was detected with anti-FLAG mAb, and GAPDH was detected as a loading control. (C) *In vitro* translation of mRNA derived from the pDluc-SHFV/WT construct or its mutant in the presence of GST-nsp1β. *In vitro* translation products were resolved in 12% SDS-PAGE and visualized by autoradiography. Products generated without frameshifting or from −1 or −2 PRF are indicated as stop, −1FS, and −2FS, respectively. (D) The expression of nsp2TF in KRCV-1 stimulated by nsp1β. HEK-293T cells were cotransfected with a plasmid expressing HA-tagged KRCV-1 nsp2 and a plasmid expressing FLAG-tagged wild-type nsp1β; empty vector (EV) was used as a control. The expression of nsp2 and nsp2TF was detected by Western blot analysis using a mAb against the HA tag, and FLAG-tagged nsp1β was detected with anti-FLAG M2 antibody. GAPDH was monitored as a loading control. (E) MARC-145 cells were infected with SHFV or mutants (vSS1, vSS2, and vCC1) at an MOI of 0.01, and cell lysates were harvested at 36 hpi. The nsp2-related products were detected by anti-PLP2 mAb 134-260. SHFV nsp2TF was also detected by rabbit pAb against the nsp2TF C-terminal peptide (nsp2TFC). GAPDH was monitored as a loading control.

As described above (Fig. 1A), the −2/−1 PRF signals (slippery sequence and C-rich RNA motif) were found in all simarteriviruses. However, four types of slippery sequence were observed in simarteriviruses of different species, namely G_GUU_UUU (SHFV), U_GUU_UUU (DeMAV, KRTGV, and PBJV), G_GUC_UCU (KKCBV, KRCV-1, KRCV-2, and MYBV-1), and U_UUC_UCU (FSVV, SHEV, and ZMbV-1). To test whether some of these variant sequences could support −2/−1 PRF in the context of the SHFV 3′ stimulatory sequence and SHFV nsp1β, plasmids pDluc-SHFV/SS1 and pDluc-SHFV/SS2 were created by introducing mutations at the SHFV slippery sequence in the dual luciferase reporter plasmid pDluc-SHFV/WT to mimic the PRF signals of distinct simarteriviruses (Fig. 6A). In the pDluc-SHFV/SS1 construct, the slippery sequence (G_GUU_UUU) was changed to G_GUC_UCU, whereas in the pDluc-SHFV/SS2 construct, the slippery sequence was changed to U_UUC_UCU (Fig. 6A). In HEK-293T cells expressing SHFV nsp1β, the SHFV/WT slippery sequence permits −2/−1 PRF, as evidenced by the detection of −1FS and −2FS expression products with mAb against *Renilla* luciferase in Western blots (Fig. 6B). In contrast, only the −2FS product was detected when the SHFV/SS1 or SHFV/SS2 construct was used. We further confirmed this result using an *in vitro* translation assay in RRL. The reporter mRNAs transcribed from pDluc-SHFV/SS1 or pDluc-SHFV/SS2 constructs and translated in RRL only generated stop and −2FS products in the presence of SHFV nsp1β (Fig. 6C).

We further confirmed the results using simarterivirus KRCV-1. As indicated above, the genome of this virus contains the slippery sequence (G_GUC_UCU). The predicted coding regions of KRCV-1 nsp1β and nsp2 were cloned into eukaryotic expression vectors. The predicted molecular masses of KRCV-1 nsp2 and nsp2TF are 77.9 kDa and 64.7 kDa. In HEK-293T cells cotransfected with plasmids containing nsp1β and nsp2, the expression of nsp2TF was detected, but no nsp2N was detected. As we expected, no frameshifting products were detected in HEK-293T cells that were not transfected with the plasmid expressing nsp1β (Fig. 6D). These data indicate that the slippery sequence of KRCV-1 lacks the ability to support −1 PRF, which is consistent with the results generated with the luciferase reporter system.

Subsequently, we investigated the function of the C-rich RNA motif in SHFV −2/−1 PRF. The plasmid pDluc-SHFV/CC1 was constructed by introducing synonymous mutations to disrupt two C-rich patches within the PRF signal (Fig. 6A). HEK-293T cells were cotransfected with pDluc-SHFV/CC1 and the plasmid expressing SHFV nsp1β. No frameshifting products were detected (Fig. 6B). This result was further confirmed by *in vitro* translation assay using RRL. Again, no frameshifting products were detected in *in vitro* translation reactions when using the reporter mRNAs transcribed from the pDluc-SHFV/CC1 construct (Fig. 6C), thus indicating that the substitutions of C residues in the C-rich RNA motif knocked out both −2 and −1 PRF.

Next, we confirmed the data from *in vitro* analysis using recombinant viruses carrying the designed mutations in pDluc-SHFV/SS1, pDluc-SHFV/SS2, and pDluc-SHFV/CC1. Using the SHFV reverse genetics system, three recombinant viruses were generated, which were designated vSS1, vSS2, and vCC1. Western blot analysis was performed to assess the expression of nsp2TF and nsp2N in WT- and mutant virus-infected MARC-145 cells. The results were consistent with the data generated in the *in vitro* expression system, in which the −1 PRF product (nsp2N) was not detected in vSS1 and vSS2, whereas expression of both −2 PRF and −1 PRF products was not detected in vCC1 (Fig. 6E). These data indicate that the C-rich RNA motif is required for efficient −2/−1 PRF in simarteriviruses and that X_XUC_UCU variants of the slippery sequence lack the ability to facilitate −1 PRF.

### Poly(rC) binding proteins are important for efficient −2/−1 PRF in simarteriviruses.

**FIG 7:**
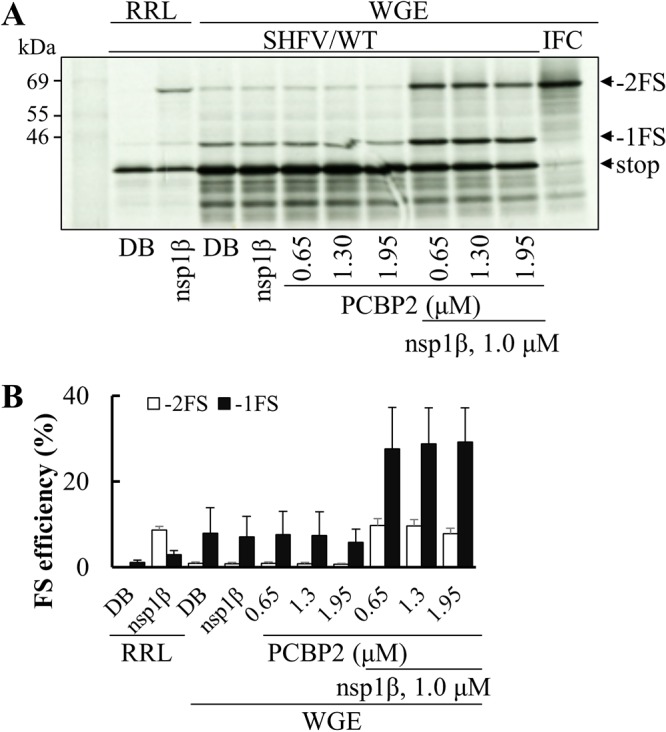
Poly(rC) binding protein 2 (PCBP2) enhances SHFV −2/−1 PRF in *in vitro* translation systems. (A) *In vitro* translation of mRNA from plasmid pDluc-SHFV/WT in wheat germ extract (WGE) in the presence of nsp1β, PCBP2, or both proteins. Translation of SHFV/WT mRNA in rabbit reticulocyte lysate (RRL) is also shown as a control. Products generated without frameshifting or from −1 or −2 PRF are indicated as stop, −1FS, and −2FS, respectively. (B) Efficiencies of −2 PRF and −1 PRF were calculated based on the quantified bands using ImageQuant TL software (GE Healthcare). Bars and error bars show mean values and standard errors of the means (SEM).

In our previous study, poly(rC) binding proteins (PCBPs) were demonstrated to be critical for −2/−1 PRF in PRRSV (35). To test whether PCBPs are also involved in stimulating −2/−1 PRF in simarteriviruses, *in vitro* translations were performed with SHFV reporter mRNA and the addition of nsp1β and/or PCBP2 in wheat germ extract (WGE). *In vitro* translation using RRL was included as the control. As expected, in RRL, both −2FS and −1FS were stimulated by the presence of nsp1β (Fig. 7A). In the WGE system, although there was some expression of −2FS and −1FS products in the absence of any exogenous protein, their levels were greatly stimulated only upon the addition of both nsp1β and PCBP2. The presence of nsp1β or PCBP2 alone showed no stimulatory effect on the translation of −2FS and −1FS products. (Note that, in contrast to RRL, WGE likely contains endogenous PCBPs that are divergent from those of mammalian cells and not active in the stimulation of PRF.) Interestingly, the frameshifting efficiencies for −1 PRF in WGE were much higher than those observed using RRL (Fig. 7B). With the addition of nsp1β and PCBP2 in WGE, the efficiency of −2 PRF increased from 0.9% to 9.8%, whereas the efficiency of −1 PRF increased from 8% to 29%. These data suggest that PCBPs are required for efficient −2/−1 PRF in simarteriviruses.

**FIG 8:**
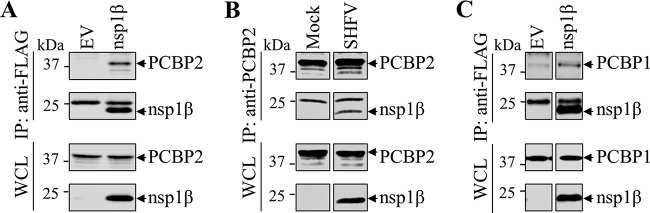
SHFV nsp1β binds to poly(rC) binding protein 1 (PCBP1) and PCBP2. (A and C) Ectopically expressed SHFV nsp1β interacts with PCBP1 and PCBP2 in HEK-293T cells. HEK-293T cells were transfected with a plasmid expressing FLAG-tagged SHFV nsp1β or empty vector. The interaction between nsp1β and PCBP1/2 was determined by immunoprecipitation (IP) using anti-FLAG M2 antibody and Western blot (WB) analysis using antibodies against PCBP1/2. (B) SHFV nsp1β interacts with PCBP2 in virus-infected MARC-145 cells. MARC-145 cells were infected with SHFV at an MOI of 0.01 and harvested at 36 hpi. The interaction between nsp1β and PCBP2 was determined by immunoprecipitation (IP) with anti-PCBP2 mAb and Western blot (WB) analysis with mAb 76-69 against SHFV nsp1β. The expression of nsp1β and PCBP1/2 was monitored by WB analysis with specific antibodies. WCL, whole-cell lysate.

Previously, the interaction between PCBPs and PRRSV-1 nsp1β was determined to be required for nsp1β’s ability to bind the PRRSV-1 −2/−1 PRF RNA signal (35). In this study, we further analyzed the interactions between PCBP1/2 and SHFV nsp1β. In HEK-293T cells transfected with plasmids expressing FLAG-tagged SHFV nsp1β and PCBP2, the protein complex of nsp1β and PCBP2 was immunoprecipitated by anti-FLAG mAb and subsequently detected by Western blot analysis using anti-PCBP2 mAb (Fig. 8A). Consistently, in SHFV-infected MARC-145 cells, nsp1β was pulled down by the mAb against PCBP2 (Fig. 8B). Similarly, the interaction between SHFV nsp1β and PCBP1 was also demonstrated by immunoprecipitation and Western blot analysis (Fig. 8C).

### The frameshift products play a role in SHFV replication *in vitro*.

**FIG 9:**
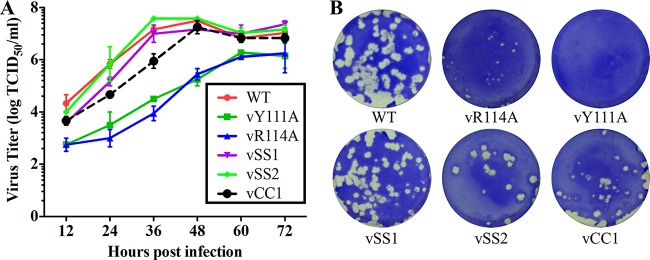
*In vitro* growth characterization of SHFV wild-type (WT) and mutant viruses. (A) Multiple-step virus growth curves of WT and mutant viruses. Each data point shown represents the mean value from duplicated treatments. Error bars show standard errors of the means (SEM). (B) Plaque morphologies of WT SHFV and mutants thereof. Conﬂuent cell monolayers were infected with 10-fold serial dilutions of the virus suspension. After 2 h of incubation, an agar overlay was added on top of the infected cells. Plaques were observed after 3 days of incubation at 37°C. Cells were stained with 0.1% crystal violet.

As described above, a panel of recombinant viruses containing mutations in nsp1β or the −2/−1 PRF signal regions was rescued using SHFV reverse genetics. Five recombinant viruses with deficiencies in the expression of nsp2N and/or nsp2TF were passaged five times (P5 viruses) in MARC-145 cells, and the introduced mutations were verified for P5 viruses (data not shown). The P3 recombinant viruses were used to evaluate viral growth kinetics *in vitro*, and WT SHFV recovered by the reverse genetics was included as the control. Growth kinetics analysis showed that vY111A, vR114A, and vCC1 attenuated viral growth in MARC-145 cells, whereas vSS1 and vSS2 displayed growth kinetics similar to that of WT virus (Fig. 9A). Before 48 h postinfection (hpi), the virus titers of vCC1 were reduced by about 1 log compared to the titer of the WT virus. In contrast, the growth of the vY111A and vR114A mutants was more significantly reduced than vCC1, with virus titers decreasing 1.5 to 3 log throughout the time course of the study (Fig. 9A). The plaque assay results consistently showed that the vCC1, vY111A, and vR114A mutants developed smaller plaques than those caused by WT virus (Fig. 9B). All three mutants displaying attenuated growth have lost the ability to express nsp2TF and nsp2N (Fig. 5 and 6E). On the other hand, vSS1 and vSS2 express nsp2TF but not nsp2N and had growth kinetics more similar to that of WT virus. This finding suggests that nsp2TF plays a role in SHFV replication, whereas nsp2N appears not to be important for viral growth in MARC-145 cells. Nonetheless, the relatively mild attenuation of vCC1, which expresses neither nsp2TF nor nps2N, confirms that neither protein is essential for viral growth in cell culture, but they may be important for maintaining maximal virus fitness.

### Heterotypic arterivirus nsp1βs stimulate ribosomal frameshifting on the SHFV −2/−1 PRF signal.

**FIG 10:**
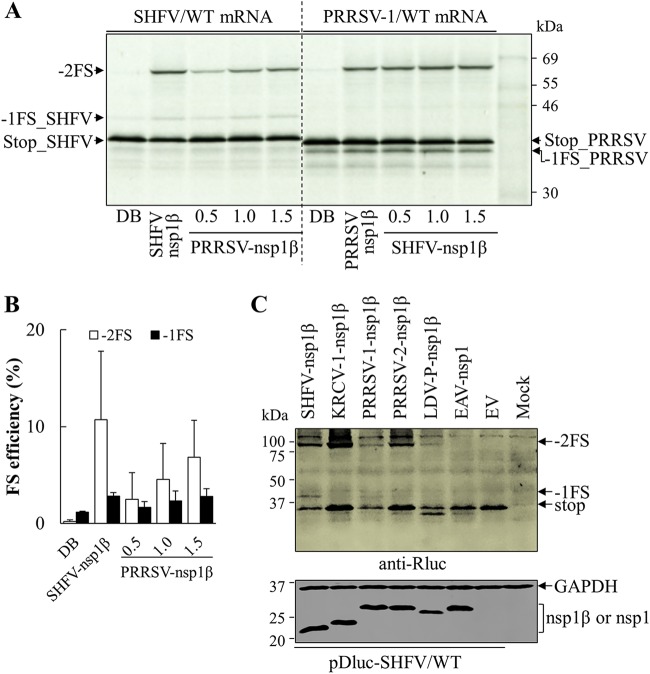
Heterotypic arterivirus nsp1βs stimulate −2/−1 PRF at signals of divergent arteriviruses. (A) Left, *in vitro* translation of mRNA from plasmid pDluc-SHFV/WT in the presence of SHFV nsp1β (1 μM), different concentrations of PRRSV nsp1β (from 0.5 μM to 1.5 μM), or dilution buffer (DB). Right, *in vitro* translation of mRNA from plasmid pDluc-PRRSV-1/WT in the presence of PRRSV nsp1β (1 μM), different concentrations of SHFV nsp1β (from 0.5 μM to 1.5 μM), or dilution buffer (DB). Products generated without PRF or from −1 or −2 PRF are indicated as stop, −1FS, and −2FS, respectively. (B) Quantification of SHFV −2 PRF and −1 PRF efficiencies when stimulated by SHFV nsp1β or PRRSV nsp1β. Frameshifting (FS) efficiencies were calculated based on the protein bands quantified by ImageQuant TL software (GE Healthcare). Each data point shown represents the mean value from two independent experiments, and error bars show standard errors of the means (SEM). (C) Analysis of −2/−1 frameshifting at the SHFV PRF signal when stimulated by heterotypic arterivirus nsp1βs. HEK-293T cells were cotransfected with the plasmid pDluc-SHFV/WT and a plasmid expressing heterotypic nsp1β. Empty vector (EV) was used as a control. Nonframeshift, −1 PRF, and −2 PRF products (indicated as stop, −1FS, and −2FS, respectively) were detected by Western blotting using anti-*Renilla* luciferase mAb. FLAG-tagged nsp1β was detected with mAb M2, and EAV nsp1 was probed with mAb 12A4. GAPDH was detected as a loading control.

The genomes of most arteriviruses (with the exception of EAV and WPDV) share a highly conserved RNA-binding motif in nsp1β, which is critical for nsp1β’s function in −2/−1 PRF transactivation (Fig. 1B). To further determine whether this binding motif is evolutionarily conserved in the *Arteriviridae* family, we evaluated the ability of PRRSV-1 nsp1β to transactivate frameshifting upon the −2/−1 PRF signal from SHFV and *vice versa. In vitro* translation was performed in the RRL system with reporter mRNA of SHFV and nsp1β protein of PRRSV-1. As expected, −2 and −1 frameshift products were detected. Increasing concentrations of PRRSV-1 nsp1β led to dose-dependent expression of the two PRF products (Fig. 10A, left, and Fig. 10B). Consistently, SHFV nsp1β displayed similar activity in stimulating −2/−1 PRF on the reporter mRNA of PRRSV-1 (Fig. 10A, right).

To confirm that the PRF transactivation mechanism of nsp1β is also conserved among other arteriviruses, we included additional heterotypic nsp1βs in the assay. The −2FS product was detected in HEK-293T cells transfected with pDluc-SHFV/WT and a plasmid expressing nsp1β from arteriviruses of other species, including KRCV-1, PRRSV-1, PRRSV-2, and LDV. Again, the −2FS product was detected; however, the −1FS product was not observed, which may be due to the low efficiency of −1 PRF (Fig. 10C). No frameshifting product was detected in cells expressing EAV nsp1. Since the canonical −2/−1 PRF signal was identified in viruses of all known arteriviral species except EAV and WPDV, these data demonstrate that the transactivation function of nsp1β on −2/−1 PRF is evolutionarily conserved in non-EAV/-WPDV arteriviruses.

## DISCUSSION

Arteriviruses are a group of mammalian positive-sense RNA viruses. Although most of them have not been associated with overt disease, some arteriviruses cause acute respiratory syndrome, abortion, lethal hemorrhagic fever, or neurological impairment (42–44). EAV, PRRSV-1, and PRRSV-2 are veterinary pathogens with significant economic impact (7). PBJV, SHEV, and SHFV are known etiologic agents of almost uniformly lethal viral hemorrhagic fever in macaques (8). Most related simarteriviruses have not been identified as pathogens, but their infectivity for and transmission ability among nonhuman primates cause concern regarding zoonotic transmission (14). Improved understanding of the biological characteristics of arteriviruses would facilitate the development of disease control strategies and may also advance our knowledge of the factors that drive zoonotic transmission of RNA viruses.

The arterivirus −2/−1 PRF and the involvement of a transactivating viral protein and host factors in ribosomal frameshifting are unprecedented in eukaryotic systems. Our recent studies revealed that the PRF products of PRRSV, nsp2TF, and nsp2N are important for viral replication. On the other hand, these proteins function as innate immune antagonists, suggesting that recombinant PRRSV with impaired nsp2TF/nsp2N expression could be developed as candidate vaccines (45). In a recent comparative genomic study, conserved +1 and −2 PRF signals were identified in additional sex combs-like (ASXL) genes 1 and 2, respectively, and hypothesized to be utilized for the expression of a conserved overlapping ORF via PRF (46). ASXL genes encode important epigenetic and transcriptional regulatory proteins, and truncation or frameshift mutants of ASXL are linked to myeloid malignancies and genetic diseases. This study highlights the significance of the −2 PRF mechanism, suggesting that the mechanism could be more widely employed in regulating viral/host gene expression.

Comparative genomic analysis of 19 arteriviruses revealed that the key elements for −2/−1 PRF, including the slippery sequence and downstream C-rich RNA motif, are highly conserved in all known arteriviruses except EAV. Of note, the distance between the slippery sequence and downstream C-rich RNA motif is consistently 9 or 10 nt in all non-EAV arteriviruses, with the exception of that of WPDV, which has a stretch that is 9 nucleotides longer. WPDV is phylogenetically distant from other arteriviruses (47). It also lacks a long overlapping TF ORF (36); thus, like EAV, WPDV most likely does not utilize frameshifting in this region of the nsp2 gene. Our experimental data indicate that a −2/−1 PRF mechanism similar to that used by PRRSV is employed by simarteriviruses to express nsp2TF and/or nsp2N analogs. In addition, we also experimentally demonstrated that nsp1β proteins of other arteriviruses (KRCV-1, PRRSV-1, PRRSV-2, and LDV, but not EAV) are able to stimulate ribosomal frameshifting on the SHFV −2/−1 PRF RNA signal. These results indicate that −2/−1 PRF is an evolutionarily conserved mechanism used by most arteriviruses for the expression of additional viral proteins. Furthermore, in line with our previous study on PRRSV, at least nsp2TF plays an important role in SHFV replication. Previous studies suggest that the N-terminal PLP2 domain shared by nsp2-related proteins of arteriviruses (PRRSV-1, PRRSV-2, LDV, and SHFV) suppresses the host type I IFN response through its deubiquitination activity (30). In comparison with nsp2, PRRSV-2 nsp2TF and nsp2N have a stronger inhibitory effect on host innate immune responses (45). Therefore, we suspect that SHFV nsp2TF and nsp2N may also play important roles in SHFV infection and virus-host interaction.

Three variants of the G_GUU_UUU slippery sequence were identified in simarteriviruses, namely, U_GUU_UUU (DeMAV, KRTGV-1, and PBJV), G_GUC_UCU (KKCBV, KRCV-1, KRCV-2, and MYBV-1), and U_UUC_UCU (FSVV, SHEV, and ZMbV-1). In the case of PRRSV, we proposed that tandem slippage of ribosome-bound tRNAs on G_GUU_UUU allows complete A site re-pairing in both −1 and −2 frames (tRNA anticodon-mRNA codon pairing in 0 frame is 3′-AAG-5′:5′-UUU-3′; single tRNA^Phe^ isoacceptor AAG). In contrast, however, tandem slippage of ribosome-bound tRNAs on G_GUC_UCU and U_UUC_UCU does not allow A site re-pairing in the −1 frame. Consistently, our results showed that the SHFV SS1 and SS2 mutants that mimic these slippery sequence variants only permit −2 PRF. Thus, those simarteriviruses carrying the slippery sequence of G_GUC_UCU or U_UUC_UCU are unlikely to be able to express nsp2N. As indicated above, the SHFV nsp2N is predicted to be an innate immune antagonist. It will be interesting to compare the pathogenicity of this group of simarteriviruses (lack of nsp2N expression) with that of SHFV (expression of nsp2N).

PRRSV nsp1β transactivates ribosomal frameshifting through a highly conserved α-helix motif, in which a universally conserved arginine (Arg128 in PRRSV-2 and Arg134 in PRRSV-1) is a key residue for nsp1β activity (34, 38). Comparative sequence analysis of nsp1β of 19 arteriviruses shows that the highly conserved α-helix motif is present in all of them except EAV and WPDV. In our study, we observed that SHFV nsp1β stimulates −2/−1 PRF to express nsp2TF and nsp2N, and an alanine substitution at Arg114 completely impairs this process. Consistently, nsp1β of KRCV-1, a newly isolated simarterivirus, is also capable of stimulating −2 PRF. Based on sequence alignment, Arg114 in SHFV nsp1β is the residue corresponding to Arg128 in PRRSV-2 and Arg134 in PRRSV-1, and this arginine is universally conserved among all arteriviruses except EAV and WPDV. Besides this arginine, Tyr111 in SHFV is also highly conserved among all arteriviruses except EAV and WPDV. Of note, alanine substitutions introduced at this residue in SHFV and at analogous residues in PRRSV-1 (Tyr131) and PRRSV-2 (Tyr125) also impair nsp1β’s ability to stimulate −2/−1 PRF of both PRRSV-1 and PRRSV-2. Remarkably, nsp1βs from PRRSV-1, PRRSV-2, KRCV-1, and LDV are capable of stimulating ribosomal frameshifting on the SHFV −2/−1 PRF signal. The amino acid sequence identities of nsp1βs among these arteriviruses range from 21.4% to 57.7%. However, *in silico* structure prediction shows that, with the exception of EAV nsp1 and WPDV nsp1β, all arteriviral nsp1βs share a similar 3-D structure, and the conserved α-helix motif is also found in these predicted structures. These data suggest that the nsp1β protein structure, especially the α-helix region, is essential for its PRF transactivation function.

Cellular PCBPs were initially identified as interacting partners of PRRSV nsp1β, nsp9, and the genomic 5′ untranslated region (5′-UTR) (48). Our recent study explored the function of PCBPs in enhancing −2/−1 PRF, together with viral protein nsp1β (35). We also observed that the enhancement of −2/−1 PRF in SHFV was dependent on the addition of both viral nsp1β and PCBP2 in WGE (Fig. 7A). Interestingly, the frameshifting efficiencies for −1 PRF in WGE were much higher than those observed using rabbit reticulocyte lysate (RRL). In our previous study on the PRRSV frameshift signal (35), we found efficient −2 PRF in RRL upon the addition of PRRSV nsp1β only. Additional supplementation with PCBP2 maintained −2 PRF but also led to an increase in −1 PRF. We reasoned that RRL may already contain endogenous PCBPs, with a balance of paralogs that favored −2 PRF over −1 PRF. Turning to WGE translations, we found that supplementation with PCBP2 led preferentially to −1 PRF, whereas supplementation with PCPB1 led preferentially to −2 PRF. This finding suggests that PCBP1 is the abundant form in RRL. Knockdown of either PCBP1 or PCBP2 in mammalian cells using small interfering RNAs (siRNAs) produced consistent results. In the current SHFV study, supplementation with PCBP2 could particularly promote −1 PRF in WGE due to the absence of competing endogenous mammalian PCBP1 (compare Fig. 7B with Fig. 4D). As we expected, the interaction between PCBP1/2 and nsp1β was detected by coimmunoprecipitation in HEK-293T cells (Fig. 8A and C). In SHFV-infected MARC-145 cells, SHFV nsp1β was also coimmunoprecipitated with endogenous PCBP2 (Fig. 8B). These data suggest that PCBPs are required for efficient −2/−1 PRF in arteriviruses, and the interaction between nsp1β and PCBPs may be also required for the mechanism.

In conclusion, our study demonstrates that −2/−1 PRF is an evolutionarily conserved mechanism used by distantly related arteriviruses for the expression of additional viral proteins, and the PRF products are important for viral replication. This mechanism is unprecedented in eukaryotic systems, not only with the efficient shift to the −2 frame, but also with the involvement of a transactivating viral protein factor (nsp1β) and host cellular protein (PCBPs). Given the crucial role of ribosome function in all living systems, the potential impact of the in-depth characterization of this novel mechanism reaches beyond the field of virology.

## MATERIALS AND METHODS

### Cells and viruses.

Embryonic grivet (Chlorocebus aethiops) kidney epithelial cells (MARC-145; ATCC CRL­12231) were used for SHFV propagation and subsequent experiments. Human embryonic kidney 293T cells (HEK-293T; ATCC CRL-3216) were used for ectopic protein expression. These cells were maintained in minimum essential medium (MEM; Thermo Fisher Scientific, Waltham, MA) supplemented with 10% heat-inactivated fetal bovine serum (FBS; Sigma-Aldrich, St. Louis, MO), 100 U/ml penicillin-streptomycin (Thermo Fisher Scientific, Waltham, MA), and 0.25 μg/ml Gibco amphotericin B (Thermo Fisher Scientific, Waltham, MA) at 37°C with 5% CO_2_. SHFV isolate KS-06_17_11 (strain LVR 42-0/M6941; GenBank accession no. KM373784.1) (49) and mutants thereof were used for experiments. These mutants were rescued with SHFV reverse genetics and designated vY111A, vR114A, vSS1 and vSS2, and vCC1.

### Plasmids.

Based on a previous study (21), SHFV nsp1β is predicted to contain amino acids (aa) 166 to 350 of ORF1a, while nsp2 contains aa 486 to 1236 of ORF1a. In this study, the coding sequences of nsp1β and nsp2 were inserted into the pcDNA 3.1^(+)^ vector (Thermo Fisher Scientific, Waltham, MA) under the control of the cytomegalovirus (CMV) promoter and were also N-terminally tagged with the FLAG (DYKDDDDK) epitope or hemagglutinin (HA) epitope, designated pFLAG-SHFV-nsp1β and pHA-SHFV-nsp2, respectively. Mutations in nsp1β were generated using the QuikChange site-directed mutagenesis kit (Agilent Technologies, Santa Clara, CA) according to the manufacturer’s instructions. Wild-type (WT) nsp1β and mutants thereof were cloned into the pGEX-6p-2 vector (GE Healthcare, Chicago, IL) for expression as glutathione *S*-transferase (GST)-tagged proteins. The coding regions of nsp1β and the PLP2 domain were cloned into the pET28a(+) vector (MilliporeSigma, Burlington, MA). Plasmids expressing nsp1β and nsp2 proteins of PRRSV were described elsewhere (34). The predicted nsp1β gene sequence of KRCV-1 (GenBank accession no. HQ845737.1) was cloned into the p3xFLAG-Myc-CMV-24 expression vector (Sigma-Aldrich, St. Louis, MO), whereas the predicted nsp2 gene sequence of KRCV-1 (GenBank accession no. HQ845737.1) tagged with an HA epitope at the N terminus was cloned into the pcDNA 3.1^(+)^ vector (Thermo Fisher Scientific, Waltham, MA). The LDV Plagemann strain (LDV-P) nsp1β gene was codon optimized for expression in human cells and cloned into the p3xFLAG-Myc-CMV-24 expression vector (Sigma-Aldrich, St. Louis, MO). The plasmid was designated pFLAG-LDV-nsp1β. The plasmid expressing nsp1 of the EAV Bucyrus strain was described previously (50). A dual luciferase reporter plasmid, pDluc (51), was used for evaluation of *in vitro* programmed ribosomal frameshifting (PRF) efficiencies. The pDluc-SHFV/WT plasmid and mutants thereof were generated by inserting the SHFV −2/−1 PRF signal into pDluc between the *Renilla* and firefly luciferase reporter genes using the method we described previously (34). The pDluc-PRRSV/WT plasmid containing −2/−1 PRF signals of PRRSV-1 was described in our previous study (34). Human PCBP2 (NM_005016.5) cloned in pcDNA 3.1^(+)^ was kindly provided by Asit K. Pattnaik, University of Nebraska—Lincoln, Lincoln, NE. For expression of the hexahistidine (His6)-tagged recombinant protein, the PCBP2 gene was cloned into the pET28a(+) vector (MilliporeSigma, Burlington, MA) with a His6 tag on the N terminus.

### Antibodies.

Monoclonal antibodies (mAbs) against SHFV nsp1β and the nsp2 N-terminal PLP2 domain were generated using the method that we described previously (52). The mouse experiment was performed according to protocols approved by the Institutional Animal Care and Use Committee (IACUC) of Kansas State University. Briefly, BALB/c laboratory mice (Jackson laboratory, Bar Harbor, ME) were immunized 3 times with 50 μg of purified protein mixed with Freund’s incomplete adjuvant (Sigma-Aldrich, St. Louis, MO) at 2-week intervals. Mouse splenocytes were fused with NS-1 myeloma cells. Specific anti-nsp1β and anti-nsp2 mAbs were obtained by screening with an immunofluorescence assay. mAbs 133-243 and 134-260 against the SHFV nsp2 N-terminal PLP2 domain and mAb 76-69 against SHFV nsp1β were used in this study. mAbs 36-19 and 140-68 were used for specifically recognizing nsp2 of PRRSV-1 and PRRSV-2, respectively (34). A polyclonal antibody (pAb) against the SHFV nsp2TF C-terminal peptide (RLDSTVVFEETTPLLDQVPVC; nsp2TFC) was produced in rabbits by GenScript (Piscataway, NJ). The following antibodies from commercial resources were also used in this study: anti-HA tag mAb 16B12 (Biolegend, San Diego, CA), anti-FLAG tag mAb M2 (Sigma-Aldrich, St. Louis, MO), anti-human PCBP2 mAb 23-G (Santa Cruz Biotechnology, Dallas, TX), anti-human PCBP1 rabbit pAb (Sigma-Aldrich, St. Louis, MO), anti-glyceraldehyde 3-phosphate dehydrogenase (GAPDH) pAb (Santa Cruz Biotechnology, Dallas, TX), and anti-*Renilla* luciferase mAb clone 1D5.2 (MilliporeSigma, Burlington, MA). mAb 12A4 against EAV nsp1 (53) was generously provided by Udeni Balasuriya at Louisiana State University.

### Protein expression and purification.

Recombinant proteins were expressed and purified using the methods described previously (35). Briefly, His-tagged nsp1β and the PLP2 domain of nsp2 were expressed in Escherichia coli BL21(DE3)pLysS (Thermo Fisher Scientific, Waltham, MA) and purified using Ni-nitrilotriacetic acid agarose resin (Ni-NTA; Qiagen, Germantown, MD). GST-tagged nsp1β and mutants thereof were expressed in E. coli BL21(DE3)pLysS (Thermo Fisher Scientific, Waltham, MA) and purified using glutathione agarose resin (Thermo Fisher Scientific, Waltham, MA). His-tagged PCBP2 was expressed in E. coli BL21(DE3)pLysS (Thermo Fisher Scientific, Waltham, MA) and purified using Ni-NTA resin (Qiagen, Germantown, MD). Proteins were dialyzed, quantified with the Pierce bicinchoninic acid (BCA) protein assay kit (Thermo Fisher Scientific, Waltham, MA), and stored at −80°C.

### *In vitro* translation.

*In vitro* translation was performed using nuclease-treated rabbit reticulocyte lysate (RRL; Promega, Madison, WI) or wheat germ extract (WGE; Promega, Madison, WI) as described previously (35). Briefly, 5′-capped messenger RNAs were generated using T7 RNA polymerase (New England Biolabs, Ipswich, MA) with the FspI-linearized pDluc reporter plasmid. *In vitro* translation reaction mixtures (10 μl) were reconstituted with mRNA template (50 μg/ml), RRL (9 μl), or WGE (9 μl) containing 20 μM amino acids without methionine (Promega, Madison, WI), 0.2 MBq [^35^S]methionine (PerkinElmer, Waltham, MA), and purified viral protein and/or cellular protein. After 1 h of incubation at 30°C, proteins were separated in 12% SDS-PAGE gels. The protein gels were dried and exposed to X-ray film or to a Cyclone plus storage phosphor screen (PerkinElmer, Waltham, MA). Images were developed using a Typhoon trio variable-mode imager (GE Healthcare, Chicago, IL), and protein bands were quantified using ImageQuant TL software (GE Healthcare, Chicago, IL). The formula (IFS1/MetFS1)/(IS/MetS + IFS1/MetFS1 + IFS2/MetFS2) was used to calculate −1 PRF efficiency. In this formula, the number of methionines in the product without a frameshift (stop) and the −1/−2 frameshift (FS) products are denoted by MetS, MetFS1 and MetFS2, respectively, and the densitometry values for the same products are denoted by IS, IFS1 and IFS2, respectively. The −2 PRF efficiency was calculated similarly. All frameshifting assays were repeated at least three times.

### Immunoprecipitation and Western blots.

Expression of PRRSV nsp2TF and nsp2N was detected by immunoprecipitation (IP) and Western blot analysis as described previously (34). Similar methods were used for detecting the expression of the SHFV nsp2-related proteins. In the ectopic expression system, HEK-293T cells in 6-well plates were cotransfected with plasmid DNA (1 μg) expressing SHFV nsp2 and plasmid DNA (0.5 μg) expressing SHFV nsp1β or its mutants. The empty vector was included as a control. At 36 h posttransfection (hpt), cell lysates were harvested with 300 μl of IP lysis wash buffer per well (Thermo Fisher Scientific, Waltham, MA) supplemented with protease inhibitor cocktail (Sigma-Aldrich, St. Louis, MO). For the SHFV infection system, MARC-145 cells seeded in 6-well plates were infected with the parental virus or mutants thereof at a multiplicity of infection (MOI) of 0.01; cells were mock inoculated to serve as a negative control. At 36 hpi, cell lysates were harvested with 300 μl of IP lysis wash buffer per well (Thermo Fisher Scientific, Waltham, MA), supplemented with protease inhibitor cocktail (Sigma-Aldrich, St. Louis, MO). IP was performed using the Pierce classic magnetic IP/co-IP kit (Thermo Fisher Scientific, Waltham, MA) according to the manufacturer’s instructions. SHFV nsp2-related proteins were immunoprecipitated with mAb 133-243 against the SHFV PLP2 domain. Western blot analysis was conducted with mAb 134-260 against the SHFV PLP2 domain and rabbit antisera against the nsp2TF C-terminal peptide. IRDye 800CW goat anti-mouse IgG (H+L) or/and IRDye 680RD goat anti-rabbit IgG (H+L) (LI-COR Biosciences, Lincoln, NE) were used as secondary antibodies. The target proteins were visualized using a digital imaging system (Odyssey infrared imaging system; LI-COR Biosciences, Lincoln, NE).

IP and Western blot analysis were also performed to determine the interactions between SHFV nsp1β and PCBP1/2, as described previously (54). In the ectopic expression system, HEK-293T cells seeded in 6-cm dishes were transfected with plasmid DNA (3 μg) expressing SHFV nsp1β. An empty vector was included as a control. At 36 hpt, cell lysates were harvested with 500 μl of IP lysis wash buffer per well (Thermo Fisher Scientific, Waltham, MA) supplemented with protease inhibitor cocktail (Sigma-Aldrich, St. Louis, MO). IP was conducted using anti-FLAG mAb M2 to precipitate the FLAG-tagged SHFV-nsp1β, and Western blot analysis was performed to detect SHFV nsp1β and PCBP1/2 using specific mAbs. For the SHFV infection system, MARC-145 cells seeded in 10-cm dishes were infected with SHFV at an MOI of 0.01. Mock-infected cells were included in the analysis as a negative control. At 36 hpi, cell lysates were harvested with 1 ml of IP lysis wash buffer per dish (Thermo Fisher Scientific, Waltham, MA), supplemented with protease inhibitor cocktail (Sigma-Aldrich, St. Louis, MO). IP was conducted using anti-PCBP2 mAb 23-G, and Western blot analysis was performed to detect SHFV nsp1β and PCBP2 with specific mAbs.

### SHFV reverse genetics system.

The SHFV infectious clone (pCMV-SHFV) consists of a commercially synthesized cDNA covering the full-length genome of SHFV (GenBank accession no. KM373784.1), with a CMV immediate early enhancer and promoter at the 5′ end and a hepatitis delta virus ribozyme sequence (pCMV-SHFV) at the 3′ end. This cassette was assembled into a pACYC177 vector backbone (GenBank accession no. X06402). The full-length cDNA clones containing mutations at the region of nsp1β or the −2/−1 PRF signal were created using the QuikChange site-directed mutagenesis kit (Agilent Technologies, Santa Clara, CA) according to the manufacturer’s instructions. Viruses were rescued by transfection of 70 to 80% confluent MARC-145 cells with 2 μg of pCMV-SHFV or mutants thereof using Transit-LT1 transfection reagent (Mirus Bi LLC, Madison, WI). Viability of the recombinant viruses was determined by observing the cytopathic effect (CPE) and by immunofluorescence assay using anti-SHFV PLP2 mAb 134-260. The cell culture supernatant was harvested at 80% CPE. The recombinant viruses were serially passaged 5 times on MARC-145 cells. Passage 5 (P5) viruses were used for subsequent study. To test the stability of mutants, substitutions in mutants of P5 viruses were verified by sequencing −2/−1 PRF signal- and nsp1β-coding regions.

### *In vitro* characterization of recombinant viruses.

Recombinant viruses were characterized by determining the viral growth kinetics. For multistep growth curves, MARC-145 cells were seeded in 24-well plates. When the cells reached 100% confluence, they were infected with parental virus or mutants thereof at an MOI of 0.01. Cell culture supernatants were collected at 12, 24, 36, 48, 60, and 72 hpi. Virus titer was measured by titration on MARC-145 cells and calculated as 50% tissue culture infective dose (TCID_50_)/ml according to the Reed and Muench method (55). To determine the plaque morphology of the parental virus and corresponding mutants, a plaque assay was performed in MARC-145 cells using the method described previously (33).
